# Acetate Metabolism in Thyroid Cancer Progression

**DOI:** 10.3390/ijms27042013

**Published:** 2026-02-20

**Authors:** Enke Baldini, Silvia Cardarelli, Eleonora Lori, Poupak Fallahi, Camilla Virili, Marco Centanni, Vito D’Andrea, Alessandro Antonelli, Salvatore Sorrenti, Salvatore Ulisse

**Affiliations:** 1 Department of Surgery, “Sapienza” University of Rome, 00185 Rome, Italy; 2 Department of Translational Research and New Technologies in Medicine and Surgery, University of Pisa, 56126 Pisa, Italy; 3 Department of Medico-Surgical Sciences and Biotechnologies, “Sapienza” University of Rome, 00185 Rome, Italy; 4 Department of Surgery, Medical and Molecular Pathology and Critical Area, University of Pisa, 56126 Pisa, Italy; 5 Interdisciplinary Department of Well-Being, Health and Environmental Sustainability, “Sapienza” University of Rome, 00185 Rome, Italy; salvatore.sorrenti@uniroma1.it

**Keywords:** thyroid cancer, acetate metabolism, prognosis

## Abstract

In recent years, several studies have highlighted the ability of malignant cells to use acetate as an alternative energy and biosynthetic source to glucose. In this context, the present study aimed at characterizing the expression profile of genes involved in acetate metabolism in thyroid carcinomas. To this end, we analyzed molecular and clinical data from 496 papillary thyroid cancers (PTCs) and 59 normal thyroid tissues from The Cancer Genome Atlas (TGCA). In addition, we examined 57 PTCs and matched normal tissues, and six anaplastic thyroid carcinomas (ATCs) collected in our institutions, using real time RT-PCR. The results show a downregulation of ACSS1, ACSS2, ACACB, PDHA1, SLC16A3 and SLC16A7 genes in PTCs compared with normal tissues, some of which were significantly lower in BRAF-mutated tumors, the more aggressive tall cell variant, and larger and/or metastatic PTCs. Overall, these findings point to a reduction in mitochondrial oxidative pathways that was more evident in advanced or aggressive disease forms. In ATCs, ACSS2 was the only upregulated gene, suggesting further tumor adaptation to the metabolic stress of rapidly growing cancers. In conclusion, our study demonstrates a dysregulated expression pattern of multiple genes involved in acetate metabolism, which could be exploited for the development of new therapeutic strategies.

## 1. Introduction

Epithelial thyroid cancers (TCs) represent the main endocrine malignancies, occurring more frequently in women than in men [1]. Most TCs are histologically differentiated (DTCs) and present as papillary (PTC) or follicular (FTC) variants, which account for approximately 90% and 5% of all TCs, respectively [2,3]. DTCs can progress to the more aggressive poorly differentiated TCs (PDTCs) and anaplastic TCs (ATCs) [2,3,4,5,6]. Although similar in many respects, TCs can display peculiar histological features, biological activities, and differentiation states as a result of specific genetic alterations [2,7]. The prognosis of DTC patients is usually excellent, with a 10-year overall survival (OS) of about 90% [8,9]. By contrast, PDTC and ATC patients are poorly or not at all responsive to ^131^I and to any available treatments, exhibiting a median OS of approximately 6 years and 3–6 months, respectively [10,11]. As recommended by the American Thyroid Association (ATA) guidelines for aggressive TCs, conventional therapies should be employed as interim strategies during molecular characterization of tumors and replaced by molecularly targeted therapies according to tumor features [5]. In this scenario, improving our knowledge of the molecular mechanisms responsible for PDTC and ATC onset and progression is paramount.

Over the last decades, it has become increasingly clear that reprogramming of cellular energy metabolism, controlled by both proto-oncogenes and tumor suppressor genes, is of primary importance in tumorigenesis to sustain the continuous cell growth and proliferation that characterize the progression of most cancers, including TCs [12,13,14].

To support the formation of new biomass, several anabolic processes are boosted to produce nucleotides, lipids and proteins in tumor cells, including TCs [15,16,17]. In normoxia, glucose and/or glutamine are the main energy sources, but in starvation or hypoxia conditions occurring within tumor masses, the acetate can become an essential fuel for cancer cell growth, required for ATP production, lipid biosynthesis, regulation of histone acetylation and, consequently, gene transcription [18,19]. In mammals, circulating acetate is primarily generated by the intestinal microbiota following the breakdown of dietary fibers [20]. The cellular uptake is likely implemented by monocarboxylate transporters (MCTs) and, in particular, by MCT1, -2 and -4, which have been shown to effectively transport acetate into cells [21]. Two enzymes catalyze the ATP-dependent ligation of acetate and coenzyme A to produce acetyl-CoA: the mitochondrial (ACSS1) and nucleocytosolic (ACSS2) acetyl-CoA synthetases [20]. Acetyl-CoA is a pivotal metabolite at the nexus between glycolysis and the tricarboxylic acid (TCA) cycle, as well as the substrate for fatty acids (FAs), lipids and steroids synthesis. Incorporation of acetyl-CoA into FAs requires carboxylation to malonyl-CoA by the acetyl-CoA Carboxylase Alpha (ACACA), followed by condensation of acetyl-CoA and/or malonyl-CoA by the FA synthase (FASN) to form long-chain FA. This enzymatic cascade relies on cytosolic acetyl-CoA.

Unlike ACACA, the acetyl-CoA Carboxylase Beta (ACACB) catalyzes the conversion of acetyl-CoA into malonyl-CoA on the outer mitochondrial membrane. Malonyl-CoA acts as a potent allosteric inhibitor of the Carnitine Palmitoyltransferase 1 (CPT-1), which controls the entry of fats into the mitochondria to be broken down for ATP production. As a result, ACACB compels cells to use energy sources alternative to fats, like glucose [22].

In mitochondria, acetyl-CoA is generated from pyruvate via pyruvate dehydrogenase (PDH) or from acetate via ACSS1. This acetyl-CoA enters the TCA cycle to generate reducing equivalents (NADH and FADH2) that fuel oxidative phosphorylation and ATP production [20]. Citrate, a TCA cycle intermediate, is exported to the cytoplasm and cleaved by ATP citrate lyase (ACLY) to regenerate cytosolic acetyl-CoA. However, metabolic stresses like hypoxia or fasting trigger inhibition of PDH, shunting pyruvate toward lactate production [23,24]. As a consequence, the TCA cycle is impaired and citrate availability is reduced, forcing proliferating cells to engage alternative sources to sustain the cytosolic acetyl-CoA pool. Indeed, ACLY depletion has been shown to inhibit the growth of highly glycolytic tumor xenografts [25]. It has been shown that ACSS2 is upregulated after ACLY depletion and is essential for the survival of cancer cells lacking ACLY [26]. Furthermore, ACSS2 was identified in a functional genomic screen as a critical enzyme for growth and survival of breast and prostate cancer cells cultured in hypoxia and low serum [27]. Depletion of ACSS2 in tumor xenografts was shown to inhibit tumor growth, and high expression of ACSS2 is frequently found in invasive ductal breast cancer (BC), triple-negative BC, glioblastoma, ovarian and renal cancers [27,28,29,30,31]. FASN is also upregulated in BC, and its increase correlates with markedly poorer prognosis [32].

Furthermore, acetylation/deacetylation events may alter the expression and function of cancer-associated proteins, significantly affecting tumor behavior. Dysregulation of acetyl-transferases, deacetylases or histone readers have been demonstrated to closely correlate with epithelial to mesenchymal transition [33].

In the present study, we sought to investigate the expression profile of genes involved in acetate metabolism in PTCs compared with normal thyroid tissues. To this end, we first searched for differentially expressed genes (DEGs) using transcriptomic data of The Cancer Genome Atlas Thyroid Cancer (TCGA-THCA) data collection. Subsequently, we analyzed DEGs by quantitative RT-PCR in our case series consisting of 57 PTCs and matched normal tissues and evaluated their possible relationships with clinical parameters and disease course. In addition, we measured the mRNA levels of these genes on a panel of six ATC-derived samples to assess whether the tumor dedifferentiation process entails variations in their expression.

## 2. Results

### Expression Levels of Genes Involved in Acetate Metabolism

First, the TCGA-THCA dataset was used to find DEGs among the key genes of acetate metabolism, namely ACACA, ACAB, ACSS1, ACSS2, ACLY, FASN and PDHA1. We also analyzed the expression levels of the monocarboxylate transporters SLC16A1 (MCT1), SLC16A7 (MCT2), SLCA16A3 (MCT3/4) and SLC16A4 (MCT4/5). SLC16A1, -7 and -3 were selected based on previous evidence that they effectively transport acetate [34,35,36]. Moreover, based on a preliminary screening of the remaining SLC16 family members in the TCGA-THCA, SLC16A4 was added to the analysis because it uniquely displayed upregulation in PTCs, whereas all other SLC16s were either downregulated or showed no significant variation.

**Figure 1:**
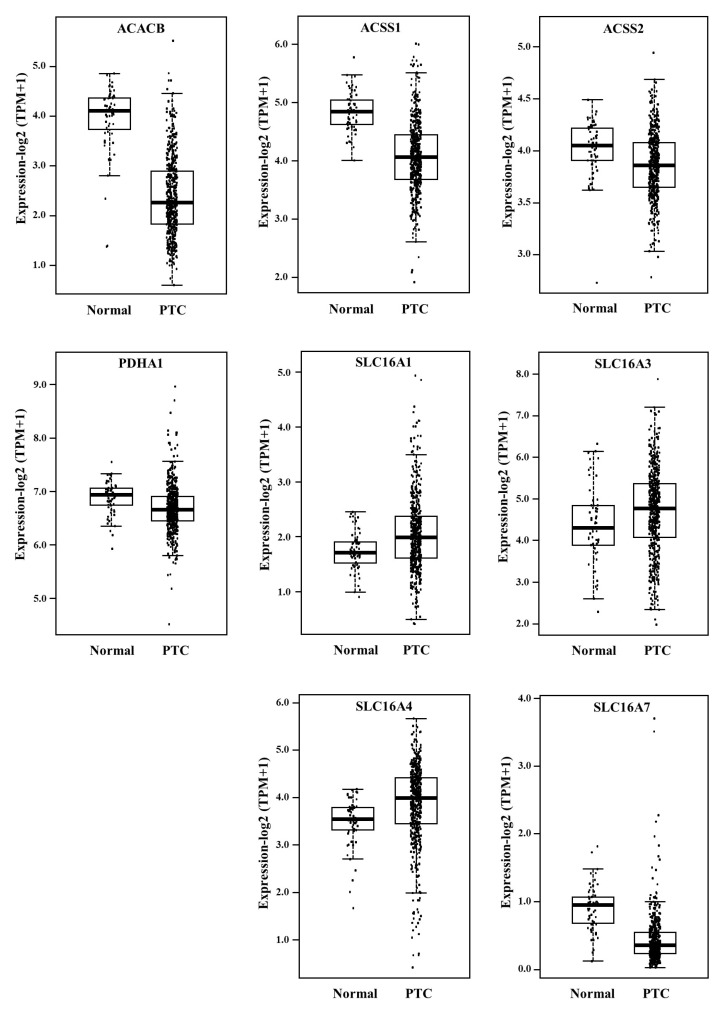
Expression levels of ACACB, ACSS1, ACSS2, SLCA16A1, SLCA16A3, SLC16A4, SLC16A7 and PDHA1 transcripts in PTC tissues compared to control tissues. Box plots of The Cancer Genome Atlas (TCGA) network case series consisting of 59 normal thyroid tissues and 496 PTC tissues. All variations were statistically significant at *p* < 0.001. TPM: Transcripts Per Million.

As shown in Figure 1, ACSS1, ACSS2, ACACB, PDHA1 and SLC16A7 mRNAs were reduced in PTCs, while SLC16A1, SLC16A3 and SLC16A4 mRNAs were increased. ACACA, ACLY and FASN mRNA levels were not significantly changed.

**Figure 2:**
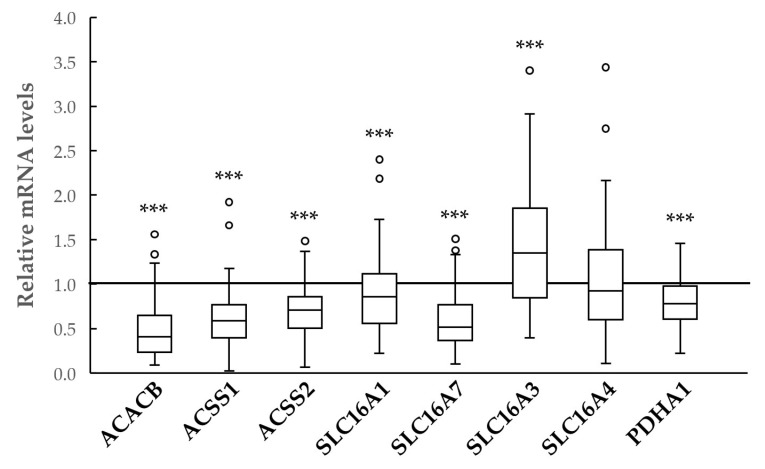
Box plot of the relative mRNA expression levels of ACACB, ACSS1, ACSS2, SLC16A1, SLC16A3, SLC16A4, SLC16A7 and PDHA1 in 57 PTCs of our case series. The horizontal line represents the values of matched normal tissues, set equal to 1. ***, *p* < 0.001.

These findings were corroborated in our case series, in which a similar expression profile was observed apart from the SLC16A4, which was unchanged, and the SLC16A1, which was downregulated (see Figure 2).

**Figure 3:**
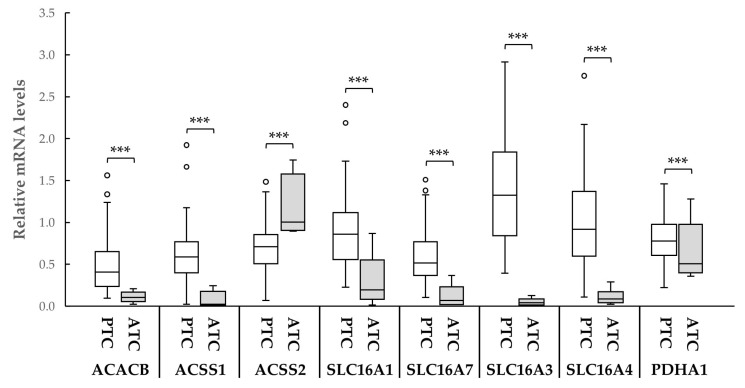
Box plots of the relative mRNA expression levels of ACACB, ACSS1, ACSS2, SLC16A1, SLC16A3, SLC16A4, SLC16A7 and PDHA1 in PTCs (n = 57) and ATCs (n = 6). ***, *p* < 0.001.

We further evaluated the expression of these genes in the six available ATC samples in comparison to PTC samples. For each mRNA, PTC values were normalized to those of matched normal tissues, while ATC values were normalized to the mean of normal tissues. As shown in Figure 3, all the genes were markedly downregulated in ATC compared with PTC except ACSS2, which was increased.

We then looked for possible correlations among the gene expression levels within the TGCA dataset. The results are shown in Table 1.

With few exceptions, the genes showed significant positive or negative correlations with each other, albeit weak or very weak. A moderate correlation was observed only between ACSS1 and ACACB.

**Figure 4:**
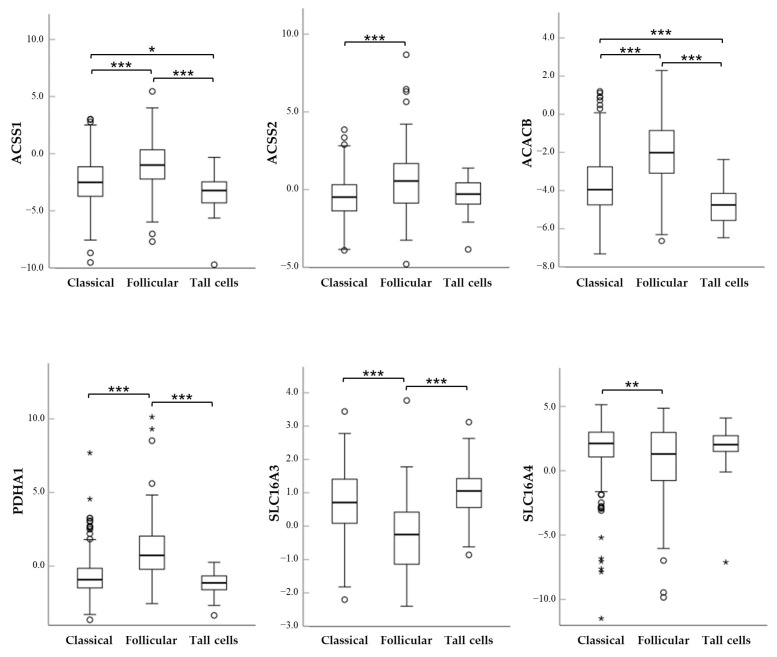
Box plots of the relative mRNA expression levels of ACACB, ACSS1, ACSS2, PDHA1, SLC16A3 and SLC16A4 in different PTC histological variants. *, *p* < 0.05; **, *p* < 0.01; ***, *p* < 0.001.

**Figure 5:**
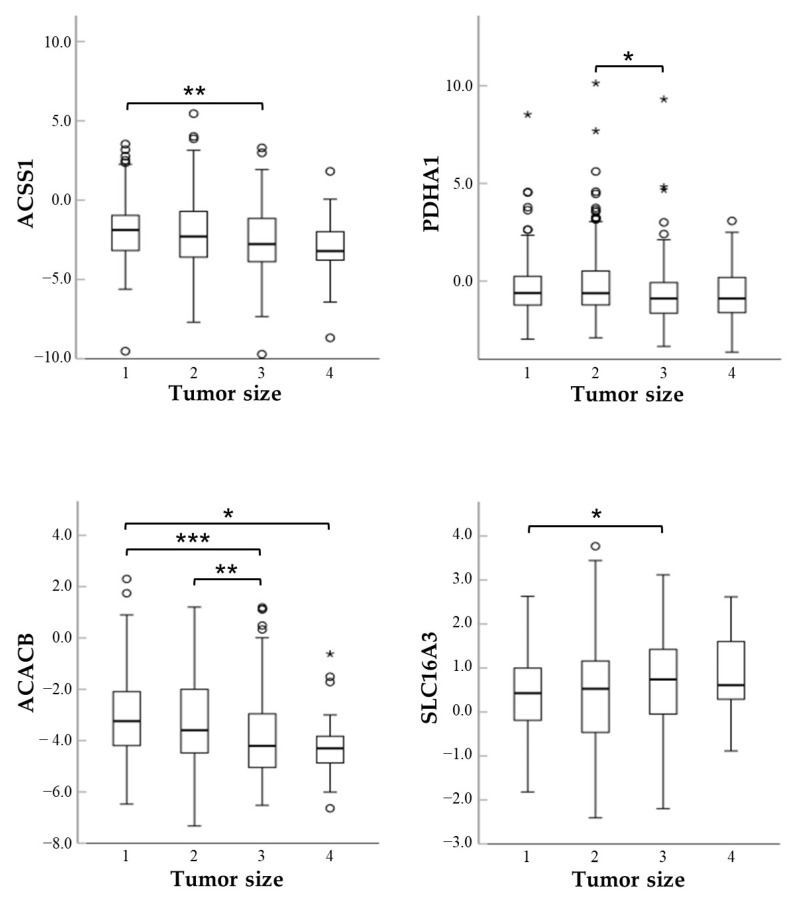
Box plots of the relative mRNA expression levels of ACACB, ACSS1, PDHA1 and SLC16A3 in the different PTC size groups. *, *p* < 0.05; **, *p* < 0.01; ***, *p* < 0.001.

Next, we performed univariate analyses to evaluate the association between gene expression levels and patients’ clinicopathological parameters, including age at diagnosis, gender, tumor histology, BRAF/RAS mutation status, thyroid differentiation score (TDS), tumor size (T), lymph node metastases (N), TNM stage, and recurrences. As noted in Table 2, none of the genes significantly associated with male or female gender. ACACB, ACSS1 and ACSS2 increased slightly with advancing age while SLC16A4 decreased. All genes except SLC16A1 and SLC16A7 were differentially expressed among the histological variants of PTCs (Table 2 and Figure 4). BRAF-like PTCs showed significantly reduced levels of ACACB, ACSS1, ACSS2, PDHA1 and SLC16A1 mRNAs but higher levels of SLC16A3 and SCL16A4 mRNAs, while that of SLC16A7 was not influenced by the BRAF status (Table 2). The expression of ACACB, ACSS1 and PDHA1 genes showed a tendency to decrease with increasing tumor size while that of SCL16A3 was significantly upregulated (Table 2 and Figure 5).

Similarly, PTCs with lymph node metastases had reduced levels of ACAB, ACSS1, ACSS2 and PDHA1 mRNAs and higher levels of the SLC16A3 and SCL16A4 mRNAs, while the expression of SLC16A1 and SLC16A7 was unaffected (Table 2). However, none of the eight genes showed significant variations between TNM stages, and PDHA1 mRNA was the only gene reduced in patients with disease recurrence (Table 2). 

Due to the smaller number of cases and the limited amount of available information, univariate analyses in our patients were feasible with fewer clinical variables. For this cohort, no relationship was observed between mRNA levels and age, sex or histological variants. Unlike TCGA patients, PDHA1 mRNA was the only one significantly reduced in patients with lymph node metastases. However, by comparing patients with T1–T2 and T3–T4 tumor sizes, we obtained results consistent with those of the TCGA dataset.

We finally created a Cox regression model to predict the probability of disease-free interval (DFI) as a function of the predictor variables reported in Table 2 and the eight mRNAs. None of the genes showed any prognostic relevance, and lymph node metastasis emerged as the only independent prognostic factor for recurrence with a hazard ratio of 8.6 (95% CI 2.1–35.0, *p* < 0.01).

## 3. Discussion

Reprogramming of energy metabolism is recognized as a hallmark of cancer cells essential to support cell growth and unrestrained proliferation [12,13,14]. In this context, enzymes involved in acetate metabolism, required for energy production, lipid biosynthesis and regulation of histone acetylation, are thought to play a role in the progression of several cancer types, and some of them are regarded as potential diagnostic or prognostic biomarkers or as therapeutic targets [21,22,23,24,25,26,27,28,29,30,31,32,33,34,35,37,38,39,40,41,42,43]. Currently, limited information is available on acetate metabolism in PTC and its clinical relevance.

The largest publicly accessible source of molecular data on PTC is the TCGA-THCA project. By analyzing transcriptomic results, we found that the expression of most genes involved in acetate metabolism was either downregulated (ACCS1, ACSS2, ACACB, PDHA1 and SLC16A7) or upregulated (SLC16A1, SLC16A3 and SLC16A4) in PTC tissues compared with control thyroid tissues. On the other hand, the transcriptional levels of ACACA, ACLY and FASN were not significantly changed. The TGCA data were largely confirmed in our case series, which displayed a similar expression profile with few exceptions.

To date, among all the genes examined, only ACACA, ACACB and SLC16A1 have been specifically investigated in thyroid tumors.

A recent study demonstrated that ACACA-depleted PTC cell lines had reduced proliferative and migratory capacities [44]. The authors identified the circular RNA circPCNXL2, overexpressed in PTCs, as a positive regulator of the ACACA activity by reducing its inhibitory phosphorylation on Ser79. High levels of circPCNXL2 boosted FA biosynthesis to support PTC cell growth both in vitro and in nude mice; conversely, circPCNXL2 knockdown inhibited tumor progression. Together with our results, this evidence suggests that, as is the case with many rate-limiting enzymes, ACACA is likely modulated at the post-translational level, allowing for rapid adjustments of the overall rate of the lipogenesis process [45].

By contrast, ACACB has been described as downregulated through histone deacetylation in acidic pH-adapted tumors [46,47]. In PTCs, reduced ACACB gene expression has been also described as a consequence of abnormal DNA methylation [48]. Both of these findings indicate that ACACB is modulated at the transcriptional level by one or more epigenetic mechanisms. A bioinformatics analysis comprising 114 PTC tissues and 126 normal tissues identified ACACB as a DEG and a hub gene whose expression was inversely related to poor overall survival rate of patients [48]. Our results showed that, besides being reduced in cancer tissues compared to normal tissues, ACACB mRNA levels were lower in BRAF-like relative to RAS-like PTCs. Similarly, a previous study reported downregulation of ACACB in BRAF^V600E^ mutated PTCs [49]. In addition, we found that the ACACB mRNA was significantly decreased in tumors of larger size, in patients with lymph node metastases, and in the tall cell PTC variant compared with the classical and follicular variants. A trend toward lower ACACB expression was also observed in higher tumor stages and in presence of recurrences. Finally, a strong ACACB reduction was noted in ATC compared with PTC tissues. Altogether, these findings support the notion that ACACB downregulation may provide a critical advantage to TC progression by enabling cells to boost FA oxidation.

The mitochondrial (ACSS1) and nucleocytosolic (ACSS2) acetyl-CoA synthetases were found to be downregulated in our PTC samples as well as in those from TGCA, with a more pronounced reduction in BRAF-like compared with RAS-like tumors. Moreover, expression of both genes was significantly lower in PTCs from patients with lymph nodes metastases. The ACSS1 mRNA was also diminished in the tall cell PTC variant compared to the classical and follicular variants. Interestingly, ACSS1 and ACSS2 had divergent behaviors in ATCs, with the former being downregulated and the latter upregulated compared with PTCs. This may be explained by considering that ACSS2 plays a prominent role in metabolic adaptation and epigenetic changes of tumor cells exposed to low oxygen levels and nutrient or lipid starvation [50]. A previous study in breast cancer cells demonstrated that ACSS2 promotes tumor cell growth under hypoxic and lipid-depleted conditions, and that ACSS2 silencing reduced tumor growth in xenograft models [27]. Similarly, ACSS2 inhibition was reported to impair proliferation, colony formation, cell motility and invasiveness of ovarian cancer cells, especially under hypoxia [30]. Unlike the slow-growing PTCs, ATCs are rapidly expanding tumors in which nutritional and oxygen restrictions are more pronounced within the tumor microenvironment, and increased ACSS2 expression may represent a crucial factor for cell survival.

Analogously to the aforementioned genes, PDHA1 expression was downregulated in PTC tissues from both case series analyzed, and to a greater extent in BRAF-like compared with RAS-like PTCs. In addition, PDHA1 mRNA was significantly lower in larger tumors, in PTC tissues of patients with lymph node metastases or disease recurrence, and also in ATC tissues. Overall, reduced PDHA1 appears to be associated with an aggressive tumor phenotype, consistent with its role in promoting aerobic glycolysis by preventing the conversion of pyruvate to acetyl-CoA.

As regards the monocarboxylate transporter genes, three of them were found overexpressed in PTC tissues of the TGCA database, namely the SLC16A1, SLC16A3 and SLCA16A4, while the SLC16A7 was under-expressed. This pattern was confirmed in our case series for SLC16A3 and SLC16A7 but not for SLC16A1 and SLC16A4, which showed reduced and unchanged mRNA levels, respectively. Such discrepancies could be explained by the smaller sample size of our patient cohort as well as the different normalization approaches employed to calculate the relative mRNA levels (i.e., comparison with matched normal tissues in our study and with unmatched normal tissues in the TGCA study) [7]. SLC16A1, SLC16A3 and SLC16A4 mRNAs were more elevated in BRAF-like PTCs, and SLC16A3 and SLC16A4 mRNAs were increased in metastatic tumors compared with non-metastatic ones. Furthermore, SLC16A3 expression was inversely correlated with the tumor differentiation score and augmented significantly with increasing tumor size. However, all the transporters were markedly downregulated in ATCs compared with PTCs. It is worth noting that a previous immunohistochemical study described SLC16A1 as highly expressed in ATCs when compared with both non-cancerous thyroid tissues and PTCs [51]. Another study demonstrated that overexpression of SLC16A1 in a PTC cell line enhanced invasive, proliferative and migratory abilities, whereas its silencing produced opposite effects [52]. These authors also reported higher SLC16A1 levels in radioiodine-refractory DTCs than in radioiodine-sensitive DTCs.

Taken together, these results are not straightforward to interpret. Although differing in substrate affinity, MCTs share the general function of transporting lactate, pyruvate and other monocarboxylates across the plasma membrane, and hence their effects could be partly redundant and/or compensatory. Nevertheless, the discrepancies encountered with respect to the TGCA data and previous works highlight the need for further analyses.

The present study suffers from some important limitations, namely the lack of information on protein expression and the small number of samples from undifferentiated tumors.

However, the concomitant downregulation of ACSS1, ACSS2, ACACB and PDHA1 suggests that PTCs undergo a metabolic shift from canonical pyruvate and acetate utilization toward increased reliance on FA β-oxidation. This change is most prominent in BRAF-like, metastatic and aggressive tall cell tumors. In ATCs, the greater reduction in PDHA1, ACSS1 and ACACB could reflect a further advancement of this metabolic transition. On the other hand, elevation of ACSS2 could represent a late-stage adaptation aimed at providing the acetyl-CoA needed for epigenetic signaling and lipid synthesis to support cell survival in a harsh tumor microenvironment.

In conclusion, our study demonstrates a dysregulated expression pattern of multiple genes involved in acetate metabolism, which likely confers an adaptive advantage to thyroid cancer cells. In particular, the results obtained provide preliminary evidence that ACSS2 inhibition could restrain ATC cell growth, though further investigations are required to fully elucidate the viability of this therapeutic approach.

A deeper knowledge of tumor-specific metabolic reprogramming could open new therapeutic scenarios, enabling the development of strategies that exploit these adaptive changes as weak points to target. Such innovative approaches are of crucial importance because, in the most aggressive and currently incurable thyroid carcinomas, effective therapy requires not only the inhibition of oncogenic drivers or hyperactivated pathways, but also the ability to overcome tumor phenotypic plasticity and treatment resistance.

## 4. Methods

### 4.1. TCGA Data

We used data from a previous study by The Cancer Genome Atlas network (TGCA-THCA) performed on 496 PTC patients [7]. The transcriptomic dataset was screened to identify DEGs among those involved in acetate metabolism, namely ACACA, ACAB, ACSS1, ACSS2, ACLY, FASN and PDHA1, together with the monocarboxylate transporters SLC16A1 (MCT1), SLC16A7 (MCT2), SLCA16A3 (MCT3/4) and SLC16A4 (MCT4/5), by using the University of Alabama at Birmingham Cancer Data Analysis Portal (UALCAN) platform [53]. For each DEG, Z-score transformed mRNA expression data of PTC samples relative to normal thyroid samples were downloaded from the cBio Cancer Genomics Portal, together with all available clinical and molecular data relating to THCA patients [54]. Transcripts Per Million (TPM) values were obtained from the Genomic Data Commons (GDC) Data Portal of the National Institute of Health. The log2(TPM + 1) transformation was applied to facilitate the comparison of fold-changes between PTCs and normal tissues across a wide dynamic range, aligning our methodology with established bioinformatic pipelines (e.g., GEO, GTEx). Z-scores were used for the correlations with clinical parameters so as to obtain a more robust identification of patients with significant deviations from the population mean.

### 4.2. Patients and Tissue Samples

Tumor and matched normal tissues were obtained from surgical specimens of 57 patients (9 males and 48 females, age range 16–76 years, median 45.5 years) who underwent total thyroidectomy for PTC at the Department of Surgery of the “Sapienza” University of Rome. Normal samples were collected from the unaffected lobe as distant as possible from the PTC mass, and the absence of tumor infiltration in the sampling area was confirmed by histological analysis. ATC tissues were collected from surgical specimens of 6 patients (1 male and 5 females, age range 57–93 years, median 71 years) who had surgery at the Department of Clinical and Experimental Medicine of Pisa. Upon harvesting, tissue fragments were quickly frozen in liquid nitrogen and stored at −80 °C until use.

All the patients enrolled in the study had thyroid hormones and TSH levels within normal ranges, and no concomitant thyroid pathologies or systemic comorbidities. Preoperative pharmacologic management was implemented in accordance with the American Thyroid Association (ATA) guidelines and the Joint Society of Anesthesiology (ESA/ASA) guidelines. Patients older than 45 years underwent total thyroidectomy with dissection of lymph nodes of the central compartment (level VI). Patients younger than 45 years underwent total thyroidectomy with central lymph node dissection only in the presence of nodal disease. Lymph node resection of the lateral neck compartments (levels II–V) was performed in patients with nodal disease diagnosed by preoperative ultrasound-guided fine-needle aspiration (FNA) cytology.

Of the 57 PTC patients, 44 (77.2%) exhibited the classical variant, and 13 (22.8%) the follicular variant. Histological diagnoses were independently performed by two histopathologists according to the World Health Organization classification [2]. Lymph node metastases were found in 20 (35.1%) patients. According to TNM (8th ed.) staging, 50 patients (87.7%) were classified as stage I, and 7 (12.3%) as stage II. Approximately two months after the initial surgery, all patients underwent radioiodine ablation followed by thyroid hormone replacement therapy. The disease-free condition was monitored 4 to 5 months later through neck ultrasound and serum Tg assay. Recurrence was diagnosed by measuring the serum Tg levels either in basal conditions or following recombinant human TSH stimulation, FNA cytology and/or Tg assay in the FNA wash-out from lymph nodes, ^131^I whole-body scan, and histological analysis following surgical resection of the lesion [55]. The follow-up was available for 39 patients (mean 51.7 ± 34.9 months, range 6–102 months), 5 of whom were at stage II and all the others were at stage I. During follow-up, 7 recurrences were recorded in cervical lymph nodes. With regard to ATC patients, all died from the disease (survival time range 1–66 months, median 5 months).

### 4.3. Extraction and Analysis of mRNA

Frozen tumor and matched normal thyroid tissues were homogenized with the Ultra-turrax, total RNA was extracted and cDNA was prepared as previously described [56]. Controls for genomic DNA contamination were carried out by omitting reverse transcriptase. The resulting templates were used for quantitative PCR amplifications of ACSS1, ACSS2, ACACB, SLC16A1, SLC16A7, SLC16A3, SLC16A4, PDHA1, and the GAPDH as an internal control, employing the LightCycler instrument (Roche Diagnostics, Mannheim, Germany), the SYBR Premix Ex Taq II (TliRNase H Plus) (Takara, Otsu, Shiga, Japan), and gene-specific primers listed in Table 3.

Amplicon specificity was first verified by automated DNA sequencing (Bio-Fab Research, Rome, Italy) and then checked by PCR melting curves. The amplification efficiency of target and reference genes was determined for each run with the LinRegPCR web application [57], and relative mRNA levels were then calculated using the Pfaffl method. These values represented the fold changes in our experimental qPCR cohorts.

### 4.4. Statistical Analysis

The Shapiro–Wilk test was applied to all continuous data, which were mostly found to have non-normal distributions. Depending on the number of categories, the Mann–Whitney U-test or the Kruskal–Wallis H-test were applied to compare the expression levels of target genes between PTC variants and tumor sizes; in BRAF^V600E^ mutated vs. BRAF wild type (BRAF^wt^) PTCs; in metastatic (N1) vs. non-metastatic (N0) PTCs; and in PTCs vs. ATCs. Bivariate correlations between mRNAs and between mRNAs and patient age or tumor differentiation score (TDS) were evaluated using the Kendall’s tau-b test. The TDS, provided by the TCGA-THCA dataset, was calculated by evaluating the mRNA expression level of sixteen genes involved in thyroid function, i.e., DIO1, DIO2, DUOX1, DUOX2, FOXE1, GLIS3, NKX2-1, PAX8, SLC26A4, SLC5A5, SLC5A8, TG, THRA, THRB, TPO and TSHR [7]. The strength of correlation was interpreted considering the following ranges of the correlation coefficient (r): 0 < r < 0.19, very weak; 0.20 < r < 0.39, weak; 0.40 < r < 0.59, moderate; 0.60 < r < 0.79, strong; 0.80 < r < 1.00, very strong. Finally, Cox regression was performed to quantify the hazard ratio (HR) of several explanatory variables, both continuous and categorical. All covariates were included in the analysis after assessment of the proportional hazard assumption and the absence of multi-collinearity. Statistics were carried out with the SPSS software version 27 (IBM, Armonk, NY, USA), and results were considered significantly different if *p* values were <0.05.

## Figures and Tables

**Table 1:** Correlation analysis of the expression levels of ACACB, ACSS1, ACSS2, SLC16A1, SLC16A3, SLC16A4, SLC16A7 and PDHA1 from the TGCA case series.

	Tau-b Kendall Correlation Coefficient							
	ACSS1	ACSS2	ACACB	SLC16A1	SLC16A7	SLC16A3	SLC16A4	PDHA1
**ACSS1**	1.000	0.313 ***p* < 0.001**	0.412 ***p* < 0.01**	−0.121 ***p* < 0.001**	−0.081 ***p* < 0.01**	−0.274 ***p* < 0.01**	−0.148 ***p* < 0.01**	0.372 ***p* < 0.001**
**ACSS2**		1.000	0.334 ***p* < 0.001**	0.030 *p* > 0.05	0.133 *p* > 0.05	−0.155 ***p* < 0.001**	−0.070 ***p* < 0.05**	0.334 ***p* < 0.001**
**ACACB**			1.000	−0.027 *p* > 0.05	0.155 ***p* < 0.001**	0.333 ***p* < 0.001**	−0.111 ***p* < 0.001**	0.294 ***p* < 0.001**
**SLC16A1**				1.000	0.194 ***p* < 0.001**	0.112 ***p* < 0.001**	−0.101 ***p* < 0.01**	−0.090 ***p* < 0.01**
**SLC16A7**					1.000	0.000 *p* > 0.05	0.032 *p* > 0.05	−0.162 ***p* < 0.001**
**SLC16A3**						1.000	−0.085 ***p* < 0.01**	−0.274 ***p* < 0.001**
**SLC16A4**							1.000	−0.127 ***p* < 0.001**
**PDHA1**								1.000

**Table 2:** Univariate analysis of ACACB, ACSS1, ACSS2, SLC16A1, SLC16A3, SLC16A4, SLC16A7 and PDHA1 expression levels and clinicopathological features of PTC patients from the TGCA dataset. In round brackets the number of patients is reported. Median values of the mRNA Z-scores in PTC tissues are listed for each category of clinical parameters. Correlation coefficients are shown for the patient age and TDS (*).

	ACACB	*p*-Value	ACSS1	*p*-Value	ACSS2	*p*-Value	SLC16A1	*p*-Value	SLC16A3	*p*-Value	SLC16A4	*p*-Value	SLC16A7	*p*-Value	PDHA1	*p*-Value
**Gender** **Male (n = 123)** **Female (n = 328)**	−3.804 −3.586	0.727	−2.388 −2.158	0.073	−0.224 −0.383	0.592	1.035 1.110	0.764	0.659 0.501	0.051	1.938 2.039	0.952	−1.539 −1.478	0.867	−0.637 −0.729	0.786
Age (yr) *	0.085	**<0.01**	0.107	**<0.001**	0.153	**<0.001**	−0.007	0.889	−0.027	0.403	−0.128	**<0.001**	0.008	0.797	0.061	0.054
**Histological variants** **Classical (n = 310)** **Follicular (n = 98)** **Tall cell (n = 34)**	−3.944 −2.011 −4.754	**<0.001**	−2.538 −0.990 −3.222	**<0.001**	−0.481 0.553 −0.285	**<0.001**	1.067 0.799 1.459	0.441	0.716 −0.252 1.055	**<0.001**	2.136 1.308 2.041	**<0.01**	−1.504 −1.405 −1.717	0.866	−0.920 0.736 −1.131	**<0.001**
**BRAF-like (n = 266)** **RAS-like (n = 116)**	−4.208 −1.300	**<0.001**	−3.804 −0.531	**<0.001**	−0.663 0.565	**<0.001**	1.146 0.673	**<0.05**	0.796 −0.390	**<0.001**	2.188 1.500	**<0.01**	−1.436 −1.196	0.227	−1.055 0.736	**<0.001**
Differentiation score *	0.576	**<0.001**	0.377	**<0.001**	0.139	**<0.001**	−0.185	**<0.001**	−0.277	**<0.001**	−0.43	0.205	0.072	**<0.05**	0.314	**<0.001**
**pT** **T_1_ (n = 130)** **T_2_ (n = 152)** **T_3_ (n = 149)** **T_4_ (n = 18)**	−3.237 −3.592 −4.204 −4.298	**<0.001**	−1.875 −2.290 −2.771 −3.205	**<0.01**	−0.304 −0.232 −0.441 −0.387	0.858	0.823 0.933 1.364 2.751	0.05	0.429 0.529 0.742 0.609	**<0.05**	2.105 2.030 1.962 1.947	0.610	−1.239 −1.239 −1.674 −1.592	0.235	−0.607 −0.616 −0.893 −0.889	**<0.05**
**pN** **N_0_ (n = 207)** **N_1_ (n = 199)**	−3.105 −4.269	**<0.001**	−1.790 −3.035	**<0.001**	−0.156 −0.671	**<0.001**	0.894 1.227	0.181	0.385 0.807	**<0.001**	1.874 2.121	**<0.05**	−1.454 −1.525	0.850	−0.496 −0.957	**<0.001**
**TNM Stage** **I (n = 366)** **II (n = 67)** **III (n = 13)** **IV (n = 4)**	−3.688 −3.953 −4.095 −4.023	0.206	−2.203 −2.771 −2.633 −2.037	0.743	−0.330 −0.336 −0.259 −0.457	0.774	0.898 1.534 2.751 −0.176	0.209	0.548 0.669 0.449 0.265	0.814	2.092 1.497 1.962 0.871	0.072	−1.466 −1.590 −1.530 −0.997	0.690	−0.638 −0.934 −0.637 −0.617	0.069
**Recurrence** **No (n = 381)** **Yes (n = 27)**	−3.776 −4.143	0.090	−2.295 −3.110	0.079	−0.417 −0.121	0.552	0.960 1.256	0.576	0.586 0.749	0.096	2.005 2.235	0.438	−1.610 −1.574	0.301	−0.635 −1.061	**<0.05**

**Table 3:** Sequences, genomic positions and amplicon sizes of the primers used in qRT-PCR for target and reference genes. ACACB, acetyl-CoA carboxylase B; ACSS1/2, acetyl-CoA synthetase 1/2; SLC16A1/3/4/7, Solute Carrier Family 16 Member 1/3/4/7; PDHA1, pyruvate dehydrogenase E1 subunit alpha 1; GAPDH, glyceraldehyde-3-phosphate dehydrogenase.

Gene	Primers	Exon	Size (bp)
ACACB	For 5′-AAGCACGACTCTGTCCTCAA-3′ Rev 5′-GCTGGCTCAGGTATATCACACA-3′	52 53	88
ACSS1	For 5′-CGATTTGTGGACGCCTACTT-3′ Rev 5′-CCCTGTGATCTGGTAATAGCC-3′	10 11	96
ACSS2	For 5′-TGCCACACCCATGAAACCC-3′ Rev 5′-CAGCTTCACCTTCCAACTCTTC-3′	13 14	98
PDHA1	For 5′-TCAAGGACAGGATGGTGAACAG-3′ Rev 5′-TCTTCCAAAGGTGGCTCAGG-3′	11 12	130
SLC16A3	For 5′-ATGGTGGCTGCGTCCTTTTG-3′ Rev 5′-AGGGCTGGAAGTTGAGTGC-3′	2 3	94
SLC16A4	For 5′-TTAGCCACCACATTTCCACTAC-3′ Rev 5′-AGCCATCCCAGCAAAGAAAC-3′	7 8	159
SLC16A7	For 5′-TGCCGTCGGACTTGTCAC-3′ Rev 5′-CCACACGCTTGCTGCTAC-3′	5 6	142
GAPDH	For 5′- ATCATCAGCAATGCCTCCTG-3′ Rev 5′- GGCCATCCACAGTCTTCTG-3′	6–7 8	136

## Data Availability

The data supporting the reported results are available on request.
